# A Mediterranean Diet May Be Protective in the Development of Diabetic Retinopathy

**DOI:** 10.3390/ijms241311145

**Published:** 2023-07-06

**Authors:** Anna Bryl, Małgorzata Mrugacz, Mariusz Falkowski, Katarzyna Zorena

**Affiliations:** 1 Department of Ophthalmology and Eye Rehabilitation, Medical University of Bialystok, 15-089 Bialystok, Poland; 2 PhD Studies, Medical University of Bialystok, 15-089 Bialystok, Poland; 3 Department of Immunobiology and Environmental Microbiology, Medical University of Gdansk, 80-211 Gdansk, Poland

**Keywords:** Mediterranean diet, diabetes, diabetes prevention, retina, diabetic retinopathy

## Abstract

The Mediterranean diet is recognized as one of the healthiest available dietary patterns. This perception results from its beneficial effects on the cardiovascular system and, also, on hypertension, diabetes, and cancer compared with other diets. Its impact on the course of diabetes is assessed in the available scientific literature; however, little information is available about its impact on diabetic retinopathy. The MD is characterized mainly by the consumption of fish, seafood, foods of plant origin, and fresh fruit and vegetables. It is also recommended to consume legumes, which are a source of folic acid, magnesium, iron, and dietary fiber. High consumption of nuts and unrefined grains is also recommended in the MD. Marine fish provide polyunsaturated acids from the omega-3 group. Olive oil plays a very important role, especially olive oil obtained from mechanical pressing. Additionally, olive oil contains vitamins E, K, and polyphenols. Polyphenols, which are present in a diverse range of vegetables, fruits, and seeds, have the ability to decrease oxidative stress, inflammation, and insulin resistance. Resveratrol is naturally found in grape skins and seeds, as well as in peanuts and berries, and is a constituent of red wine. Resveratrol can inhibit increased vascular leakage and loss of pericytes and regulate the level of VEGF protein in the retina, thus inhibiting the development of DR. Consumption of fruits, vegetables, fish, and olive oil may be correlated with a lower risk of diabetic retinopathy. This paper presents the definition of the Mediterranean diet and its influence on the course of diabetes and diabetic retinopathy.

## 1. Introduction

Diabetes mellitus (DM) and its complications constitute a dire social problem. According to the International Diabetes Federation, the worldwide prevalence of DM in 2019 was 9.3% (463 million) among people between the ages of 20 to 79. This number is predicted to steadily increase. In 2045, about 10.9% (700.2 million) of people will suffer from DM [1,2]. Both type 1 (T1DM) and type 2 (T2DM) diabetes are associated with vascular complications that can be divided into macro- and microvascular [3]. Macrovascular lesions may result in atherosclerosis and cerebrovascular incidents, cardiovascular disorders, and heart failure, while microvascular damage mainly results in renal injury, retinal complications, or neuropathy [3]. The duration of DM, glycemic control, arterial hypertension, creatinine levels, and low-density lipoprotein (LDL) cholesterol are important factors in the development of diabetic retinopathy (DR) [4,5]. It has been shown that lack of physical activity and eating habits have a large impact on the risk of diabetes and its ophthalmic complications [6,7].

The Mediterranean diet (MD) is currently recognized as the best dietary pattern. The concept of the MD diet was introduced by Ancel Keys, who observed in the Seven Countries Study that the eating habits of some Mediterranean populations are the main cause of low rates of cardiovascular disease and cancer in these communities in comparison to other studied populations [8]. The MD is characterized mainly by the consumption of fish, seafood, and foods of plant origin. These are fruits and vegetables, bread and cereals (especially whole grains), beans and other legumes, and nuts. Olive oil is the main source of fat. Dairy products (cheese and yogurt) are consumed daily in moderate amounts. Red meat is eaten occasionally, and a glass of wine is usually consumed during the meal [9]. 

In our manuscript, we decided to analyze the current scientific literature with regard to the effects of following the MD on the development and course of DR. Peer-reviewed journals were the main source of all articles we have considered in our paper. Pertinent articles were initially found in ScienceDirect, Web of Science, and PubMed databases. Filters were set to full-text, peer-reviewed articles published between 2000 and 2023 in English. We used the following keywords in our search: Mediterranean diet, diabetes, diabetes prevention, retina, and diabetic retinopathy. After applying these filters, 1036 articles were found, which were first evaluated by title and abstract. Articles whose title or abstract met these criteria were then reviewed by checking their full text. Ultimately, 140 articles were included. All authors agreed to include the articles. After reviewing the available literature, we included all relevant information in our work.

## 2. Diabetic Retinopathy (DR)

Diabetic retinopathy (DR) is a complication that poses a risk to every person suffering from diabetes. The key factors that lead to its development are hyperglycemia, hypertension, and the duration of diabetes mellitus. Due to the increasing incidence of DM and the aging of the general public, DR has become the leading cause of blindness in the working population. It is estimated to be responsible for 2.4 million cases worldwide: 15–17% in the US and Europe and 3–7% in Southeast Asia and the Western Pacific [10,11]. Non-proliferative diabetic retinopathy (NPDR) and proliferative diabetic retinopathy (PDR) are both clinical forms of diabetic retinopathy. NPDR is the initial stage of diabetic retinopathy. Its symptoms include severe retinal vascular permeability, formation of micro-aneurysms, exudation, hard exudates, and hemorrhage. Neurosensory retinal atrophy and ischemia, under such conditions, might lead to severe vision loss. PDR is in the advanced stage. It is manifested by nerve fiber layer infarcts, neovascular proliferation, and hemorrhage. Visual impairment can be exacerbated by the detachment of the retina due to traction and further bleeding [12].

**Figure 1:**
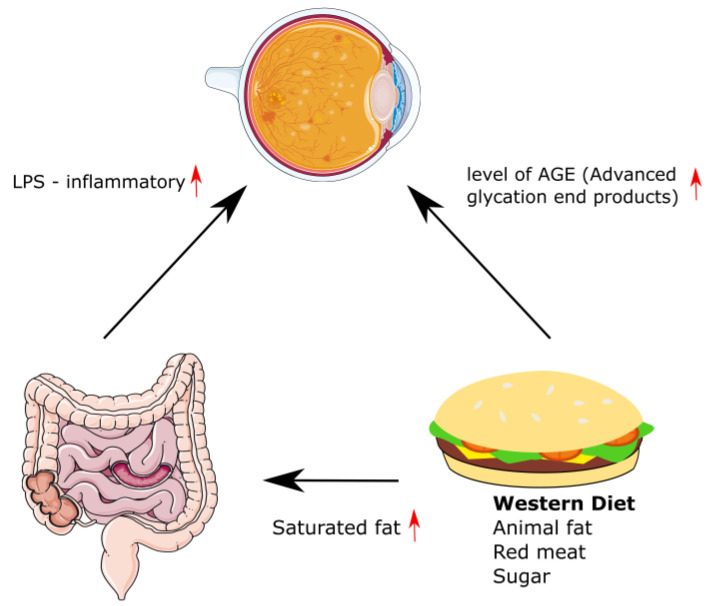
The influence of the Western diet on the development of diabetic retinopathy. This image was made using photos from Servier Medical Art (Creative Commons Attribution 3.0). Red arrows in the figure indicate an increase in level.

The International Diabetes Federation (IDF) estimated that the global population with diabetes mellitus (DM) reached 463 million in 2019 and projected it to be 700 million by 2045 [1,2,13]. At the time of diagnosis, DR is not found in T1DM patients. It develops at a later stage, and as many as 99% of diabetic patients may show a variety of its symptoms after 20 years of suffering from the disease. DR may already be present, however, in T2DM patients on diabetes diagnosis. Approximately 60% of patients suffering from type 2 diabetes show symptoms of retinopathy 20 years after the initial diagnosis [14]. Western diet significantly increases retinal leukocyte accumulation and endothelial death in the course of diabetes [7,12,15]. The influence of the Western diet on the development of diabetic retinopathy is shown in Figure 1.

Regulation of HbA1c and blood pressure is the primary goal of DR management. However, reducing serum lipid levels and fighting obesity are among other concerns [16,17,18]. These goals may be accomplished through prophylactic examinations, thus achieving earlier diagnosis and tailoring the therapy to individual patients. Promoting a healthy lifestyle may be the course of action that impacts the course of the disease in a significantly positive way. 

## 3. Mediterranean Diet in the Course of Diabetes

The MD is characterized mainly by the consumption of fish, seafood, foods of plant origin, and fresh fruit and vegetables [19]. Milk and dairy products (low-fat cheese, skimmed milk, yogurt) are sources of protein [18,19,20]. Ibsen et al. suggested that the substitution of whole-fat yogurt for milk among those aged 56–59 decreases the risk of type 2 diabetes, and the substitution of skimmed milk for semi-skimmed milk may increase the risk among those aged 60–64 and 65–72 [18]. The higher protein intake was able to ameliorate glycemic control and hyperglycemia. This was achieved without pharmacological intervention in diabetics and prediabetics [19,20]. Moreover, in the MD, milk is often replaced with yogurt, kefir, buttermilk, and cottage cheese. Feta cheese is especially recommended. It is made of 70% sheep’s milk and 30% goat’s milk and can be used in salads or as an ingredient in soups [20]. On the other hand, the consumption of red meat should be low or even occasional. White meat (chicken, turkey, rabbit) is recommended. A characteristic feature of the diet is the low consumption of animal fat (lard, butter) [21,22,23,24]. The MD involves water consumption at a level of approximately two liters per day. Vegetable and fruit juices are limited. Meals should be prepared using fat-free methods. Salt is limited to 5–6 g per day. Depending on the region, natural spices, such as rosemary, basil, oregano, turmeric, or thyme, are used to prepare dishes [20,25]. The Mediterranean diet involves low consumption of sweets. Moderate consumption of ethanol, mainly red wine, together with main meals is acceptable, with wine usually consumed with main meals [26,27]. By its very nature, the MD is not uniform. Various regions in the Mediterranean basin have their own dietary habits of different parameters. For example, the total daily lipid intake can be as high as in Greece (~40% of total energy consumption) or as moderate as in Italy (~30% of total energy consumption), but in both cases, monounsaturated fats are the main source of dietary lipids [28]. Pork and wine are rejected by Muslim countries. It has been observed that consumption of extra virgin olive oil is low in Middle Eastern countries and North Africa as opposed to Spain, Italy, or Greece. Italy is widely known for its popularity and appreciation of pasta, which is a key component of the Mediterranean diet. Those differences can have an impact on the risk of DM occurring in the course of DR; however, we focus our review on the classic definition of the MD [29].

### 3.1. Mediterranean Diet and Biochemical Parameters in T1DM Patients

The scientific inquiry into the influence of the MD on the course of type 1 diabetes is limited. The American Diabetes Association refers to the MD as a dietary model that both adults suffering from diabetes and minors suffering from T1DM should follow [30,31]. Zhong et al. found that patients with higher KIDMED (Mediterranean Diet Quality Index) scores had lower HbA1c, lower total and LDL cholesterol levels, and higher HDL cholesterol levels [32]. Cadario et al. proved that in children suffering from T1DM, the MD had a beneficial effect not only on the cardiovascular system, LDL and HDL cholesterol levels but also on interprandial glycemia [31]. Dominguez-Riscart J et al. also observed an improvement in HbA1c in the MD-adhering group. However, they did not find any differences in the lipid profile between the groups [33]. The European Childhood Diabetes Registers (EURODIAB) Prospective Complications Study conducted studies on the effects of saturated fatty acid (SFA) and dietary fibers on the onset of cardiovascular diseases [34]. The observational data obtained demonstrate a negative association between the consumption of dietary fiber and the risk of cardiovascular diseases among these patients [35,36].

### 3.2. Mediterranean Diet and Lower Risk of Type 2 Diabetes

The ATTICA study, conducted on the inhabitants of the Attica province in Greece, has demonstrated that adherence to the MD was inversely correlated with the risk of T2DM [37]. The Di@bet.es study conducted on 5076 people from Spain did not show similar dependencies [38]. A prospective study involving 8291 Italian patients with previous myocardial infarction who did not have T2DM at the start of the study showed that participants who adhered to the MD had a 35% lower risk of developing T2DM in comparison with those who did not adhere to the diet [39]. Martinez-Gonzalez et al. conducted a study on a large group of Spaniards aged 20–90. The subjects did not suffer from T2DM at the beginning of the study, and the follow-up was 4.4 years. They assessed their adherence to the MD using a questionnaire on a 9-point scale, where 0 meant no use of orders, and 9 had very good adherence to the diet. MD adherent participants with a score of MDS > 6 had an 83% reduced risk of developing T2DM in comparison with adherent participants whose score was MDS < 3. The authors also observed that a 2-unit increase in MDS score was associated with a 35% reduction in the risk of developing T2DM [25]. De Koning et al. obtained similar results [40]. They conducted a prospective study on a large number of men who were initially free from T2DM, cardiovascular disease, and cancer. The study duration was ≤20 years. Participants who adhered to the MD (score > 6) had a 25% reduced risk of developing T2DM compared with those who did not (score < 3). The reduction in the risk of T2DM was greater in overweight or obese participants in comparison with non-obese participants [40]. Abiemo et al. conducted a study involving 5390 people initially not suffering from T2DM. The duration of the study was approximately 6.6 years. Participants who adhered to the MD (score > 6) had lower baseline glucose and insulin levels compared with those who did not (score < 4). However, they did not show a significant relationship between MD adherence and the risk of developing T2DM [41]. Rossi et al. demonstrated that MD-adherent participants (MDS > 5) had a 12% reduced risk of developing T2DM compared with non-adherent participants (MDS < 4). However, adherence to MD was inversely associated with the risk of T2DM only in overweight participants [42].

Most of the studies discussed previously show a beneficial effect of the MD on glycemic control and insulin sensitivity in T2DM patients compareds with other diets [43]. A beneficial effect of the MD on cardiovascular disease risk factors (body mass index, waist circumference, blood lipids, blood pressure, inflammatory markers, and adhesive molecules) has also been demonstrated in patients with T2DM [27]. Ciccarone et al. conducted a comparison of T2DM patients suffering from peripheral arterial disease with T2DM patients with no macrovascular complications.

Regardless of the duration of diabetes and the presence of hypertension, individuals with a higher MD score had a 56% reduced probability of developing peripheral arterial disease [44]. A lower risk of subsequent cardiovascular events after myocardial ischemia has also been demonstrated in patients following MD recommendations, as well as a reduction in the total and CVD-related risk of mortality per 1 unit of increase in the MD score [27,45,46].

## 4. Natural Food as Anti-Inflammatory Ingredient for Diabetic Retinopathy

### 4.1. Foods and Nutrients in the Course of Diabetes and Diabetic Retinopathy

**Figure 2:**
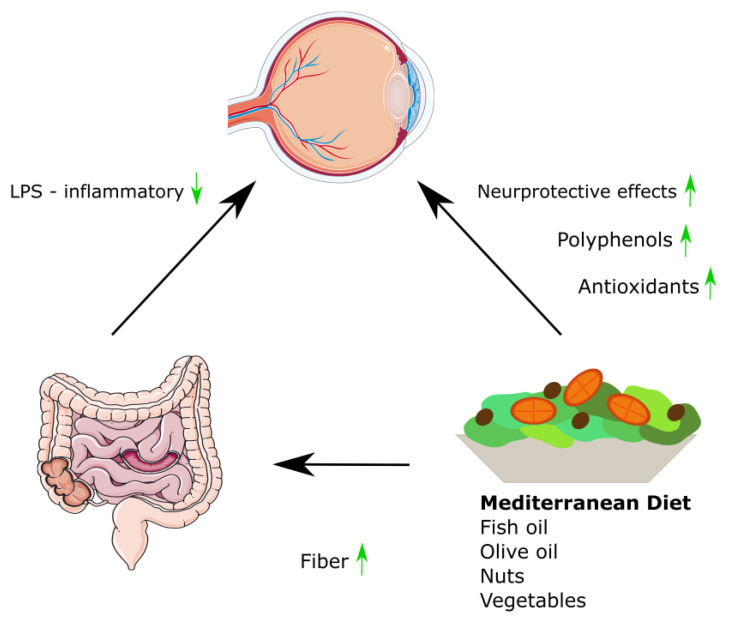
The positive influence of the Mediterranean diet on eyesight in the course of diabetes. Figures were made using pictures from Servier Medical Art (Creative Commons Attribution 3.0). Up arrow indicates growth, down arrow indicates decrease.

Oxidative stress and inflammation are reduced by foods that comprise the MD [47,48,49]. They also help decrease the pathogenic factors in diabetes and diabetic retinopathy—insulin resistance and secretion [50,51,52]. Polyphenols and other phytochemicals are found in a variety of vegetables, fruits, and seeds and can reduce oxidative stress, inflammation, and insulin resistance [53]. Thus, consumption of flavonoid-rich fruits and vegetables is correlated with a lower risk of diabetic retinopathy [54]. In a study conducted by Ghaemi et al., it was discovered that patients with both type 1 diabetes (T1DM) and type 2 diabetes (T2DM) who adhered to the MD exhibited a significantly reduced risk of cardiovascular disease (CVD) and microvascular complications associated with diabetes, such as diabetic retinopathy, nephropathy, and neuropathy, in comparison to patients who did not follow the recommended MD guidelines [55]. The positive influence of the MD on eyesight in the course of diabetes is shown in Figure 2.

#### 4.1.1. Olive Oil

Oleic acid is the basic ingredient in olive oil. It is a monounsaturated fatty acid. Additionally, olive oil contains vitamins E, K, and polyphenols [19,54,55]. A study conducted by Diaz-Lopez et al. involved 3600 participants with type 2 diabetes who were free of microvascular complications and who followed a Mediterranean diet supplemented with either extra virgin olive oil or nuts. During a median follow-up of 6.0 years, the authors identified 74 new cases of retinopathy and 168 of nephropathy. Compared with the control diet, multivariable-adjusted HRs for diabetic retinopathy were 0.56 (95% CI 0.32–0.97) for the MedDiet + EVOO and 0.63 (0.35–1.11) for the MedDiet + Nuts. No between-group differences were found for nephropathy. When the yearly updated information on adherence to the MedDiet was considered, the HR for retinopathy in the highest versus the lowest quintile was 0.34 (0.13–0.89; *p* = 0.001 for trend) [54]. In addition, those who adhered to olive oil-enriched MD had more than 60% lower risk of retinopathy compared with those who were following this diet imperfectly [54]. The way individual fatty acids affect DR is discussed in Section 4.3 Fat and Fatty Acids.

#### 4.1.2. Nuts

Nuts are characterized by a high energy value and are rich in microelements and bioactive compounds. It has been shown that they play an important role in protecting against obesity and supporting weight loss [19,21]. Nuts increase satiety and participate in lipid oxidation and thermogenesis [19,21]. The MD enriched with nuts was associated with a 37% reduction in the risk of diabetic retinopathy [54]. Peanuts contain resveratrol (RSV), which can be characterized as a polyphenolic phytoalexin in the stilbene family. RSV can modulate intracellular enzymes, such as kinases, lipoxygenases, cyclooxygenases, and free radical scavengers [56]. Therefore, it exhibits anti-glycation, anti-inflammatory, antioxidant, neuroprotective, and anticancer effects [57]. RSV treatment inhibits increased vascular leakage and loss of pericytes and regulates the level of VEGF protein in mouse retinas, thus inhibiting the development of DR [58]. It was also shown that RSV-treated retinal pigment epithelium cells significantly inhibited the secretion of VEGF, TGF-β1, cyclooxygenase-2 (COX-2), IL-6, and IL-8 in a dose-dependent manner [59]. 

#### 4.1.3. Fruits and Vegetables

Fruits and vegetables constitute an important source of vitamins, minerals, fiber, and flavonoids [60]. Consuming a high amount of fruit, equivalent to approximately 173.2 g per day (such as a large apple or two bananas), has been linked to a more than 50% decreased risk of diabetic retinopathy (DR) compared with individuals who consumed less than 53.2 g of fruit per day [52,61]. Grapes, and more precisely, their skin, contain large amounts of the aforementioned RSV. RSV is also found in apples, blueberries, and cranberries [62,63]. Spinach, broccoli, tomatoes, green peas, and Brussels sprouts all contain α-lipoic acid. α-Lipoic acid, a biological antioxidant, has the potential for reactive oxygen species (ROS) scavenging [64]. It prevents the accumulation of oxidatively modified DNA and diabetes-induced increase in nitrotyrosine levels. α-Lipoic acid decreases retinal mitochondrial and cytosolic ratios of oxidized forms and reduces nicotinamide adenine dinucleotide (NAD + and NADH). Activation of NF-κB and decreased VEGF levels and oxidatively modified proteins in the retina are also prevented by this acid [65,66,67]. Citrus fruits, blueberries, kiwi, tomatoes, green leafy vegetables, cauliflower, broccoli, red and green cabbage, and Brussels sprouts are all sources of vitamin C [67]. Carrots, green leafy vegetables, pumpkins, papaya, prunes, and peaches contain large amounts of vitamin A [67]. Carrots, green leafy vegetables, pumpkin, broccoli, peas, peaches, prunes, and apricots contain large amounts of β-carotene. Tomatoes are rich in lycopene, as are asparagus and grapefruit. Lycopene is a carotenoid that can influence the activity of lipoxygenase and thus modulate inflammation and immunological function. It has been shown to increase antioxidant activity in the eye capillaries [18]. Significantly lower levels of lycopene in the serum were observed in people with advanced stages of DR [68]. There is lutein/zeaxanthin in green leafy vegetables, lettuce, broccoli, Brussels sprouts, and string beans [67]. Spinach, avocados, and apricots are sources of vitamin B6 [67].

### 4.2. The Role of Fiber in the Mediterranean Diet in the Course of Diabetes

Fiber is an important element of the diet and can be found in legumes, whole grains, fruits, and vegetables [69]. Fiber-rich complex carbohydrates should constitute at least half of the daily energy intake of diabetic patients; a low-carbohydrate diet is recommended [70]. Dietary fibers make carbohydrate absorption time in the upper jejunum shorter, and they decrease the insulin demand, thus leading to quicker intestinal transit [17,71]. After ingestion, dietary fibers slow down glucose response, improve diabetic dyslipidemia, suppress low-grade systemic inflammation, and lower blood pressure [61,67,72]. However, scientists differ in their opinions regarding the influence of fiber on the development of DR [61,68]. A study of subjects with T2DM from the general population in India found that low fiber consumption was associated with a 41% increased likelihood of DR [68].

### 4.3. Fat and Fatty Acids

It has been shown that total fat consumption was not associated with the risk of DR [73,74]. However, there are conflicting results for particular groups of fatty acids [52]. Alcubierre et al. found that total consumption of saturated fatty acids (SFA) and individual consumption of SFA (palmitic or stearic acid) was not associated with the risk of DR [74]. On the other hand, Sasaki et al. conducted a study with participants with well-controlled T1DM and T2DM (HbA1c 7.0%) and reported an increase in the risk of DR corresponding to increasing consumption of SFA [73]. The results regarding the consumption of monounsaturated fatty acids (MUFA) are also divergent. Sasaki et al. did not find any link between retinopathy and the factors studied, whereas Alcubierre et al. discovered that a higher intake of monounsaturated fatty acids (MUFA) and oleic acid was connected to a reduced risk of developing retinopathy [74]. Alcubierre et al. found no association between DR and consumption of trans fats or polyunsaturated fatty acids (PUFA), including omega-3 PUFA (n-3 PUFA) and omega-6 PUFA (n-6 PUFA) [74]. Sasaki et al. [73] noticed a positive relationship between PUFA consumption, although only in people with well-controlled diabetes (HbA1c 7.0%). Sala-Vila et al. demonstrated that participants consuming N-3 PUFA for cardiovascular prophylaxis (500 mg/day) had a lower risk of DR [75]. People on a modified fat diet rich in linoleic acid suffered from DR less often than those on a low-carbohydrate diet and those who did not comply with recommendations, although the difference was not statistically significant [69]. Howard-Williams et al. [69] similarly noted an increased occurrence of diabetic retinopathy in individuals with inadequately controlled diabetes (HbA1c ≥ 8%) and lower levels of linoleic acid [69,72]. Alcubierre et al. [74] did not observe a correlation between linoleic acid intake and DR. Another study confirmed the protective effects of omega-3 fatty acids in proliferative diabetic retinopathy by reducing neovascularization and improving diabetic macular edema [76]. Similar results were obtained by Rosenberg [77].

### 4.4. Fish

The consumption of fish inhibits the development of DR [53]. Eating mainly fatty fish is recommended. It was found that consuming fish at least twice a week was associated with an almost 60% reduction in the risk of DR [52,71]. Another study showed that weekly consumption of 85 to 141 g of dark meat fish (salmon, mackerel, swordfish, sardines, or bluefish) was associated with a 70% reduction in the risk of DR [78]. The PREDIMED study found that omega-3 fatty acids from fish in the MD may reduce the risk of DR due to their anti-inflammatory effects [71,79,80]. In middle-aged and elderly people suffering from T2DM, the consumption of at least 500 mg of linoleic acid per day, easily achievable with 2 weekly servings of fatty fish, is associated with a reduced risk of eyesight-threatening DR [81]. Mayor was able to obtain similar results [82]. Yee et al. have shown that marine fatty acids have been demonstrated to protect against diabetic neuropathy [83]. An interesting study was conducted by the team of Alsbirk et al. [84]. The authors included 50 patients with type 1 diabetes and 460 patients with type 2 diabetes. All patients had diabetes for ≥1 year. Participants’ self-reported information regarding medication, diet supplements, HbA1c levels, and fish consumption was recorded. In the group with type 1 diabetes (T1DM), the average number of fish meals per week was 3.2, with a range of 0 to 10 meals. Among these, they consumed an average of 1.6 (0–4) warm fish meals and 1.6 (0–7) cold fish meals per week. The older group with type 2 diabetes (T2DM) had an average of 4.4 fish meals per week, including 2 warm fish meals and 2.4 cold fish meals per week. Additionally, 44% of T1DM patients and 55% of T2DM patients regularly took omega-3 supplements. The average weekly intake of omega-3 supplements was 3 for T1DM patients and 3.6 for T2DM patients, with a range of 0–7. When considering the combined intake of fish meals and/or omega-3 supplements per week, the average was 6.2 for T1DM patients and 7.9 for T2DM patients. The average weighted weekly intake of fish oil was 6.5 for T1DM patients and 8.1 for T2DM patients. Importantly, none of the 510 patients experienced visual acuity worse than 0.3 due to diabetic retinopathy, indicating a low rate of visual impairment compared with similar studies [84]. The potential protective effect of polyunsaturated fatty acids (PUFA) on the prevalence and progression of diabetic microangiopathy, including retinopathy, is discussed [85]. Notably, in Japan, where fish consumption is known to be up to five times higher than in Western countries, the incidence and progression rate of diabetic retinopathy appear to be lower than in Western populations [86].

## 5. The Influence of Selected Micro- and Macroelements in the Mediterranean Diet and Diabetic Retinopathy

Two studies on people suffering from T2DM and a study on people suffering from T1DM have associated neither potassium nor sodium intake with a risk of DR [87,88]. Studies on the influence of carotenoids on the risk of DR are not consistent [61,89,90]. Brazionisa et al. noted that similarly to AMD, increased levels of lutein and zeaxanthin were associated with a significantly lower risk of DR [91]. Lutein has been shown by several studies to delay DR progression within 5 years [92]. Sensitivity to contrast, glare, and visual acuity in patients with non-proliferative DR have all been improved by daily applications of 10 mg of lutein [93]. Sahli et al. have not found any relationship between lutein consumption and retinopathy [90]. Similarly, Mayer-Davis et al. have not shown a relationship between the consumption of β-carotene and DR [89]. Tanaka et al. obtained different results [61]. The results regarding the effects of vitamin C on DR are also inconsistent. The authors found that high vitamin C intake is associated with a 40% reduced risk of retinopathy [61], while other researchers have not shown a similar relationship [94,95]. Oral vitamin C has been shown in human and animal studies to decrease dysfunction of the capillary endothelium in those suffering from diabetes [95]. Proliferative diabetic retinopathy leads to a significant reduction, approximately tenfold, in the concentration of ascorbate in the vitreous humor. Moreover, it is associated with an elevated susceptibility to diabetic macular edema [96]. Vitamin C, together with statins, reduces the effects of non-proliferative DR, in a dose-dependent manner, to a greater extent than statins alone [97]. A Joslin Institute study demonstrated that in patients suffering from T1DM for less than 10 years, a dose of 1800 IU daily vitamin E improved retinal blood flow [98]. Treatment with vitamin E reduces oxidative stress, which is typically elevated in DR [99]. Vitamin E administered with vitamin C appears to have more beneficial effects [100]. Vitamin D is necessary for arterial stiffness, reduction in inflammation, insulin release, and sensitivity to it [101]. It has been shown that optimal levels of vitamin D are necessary for the reducing risk and severity of DR [102]. Vitamin D plays an important role in the functioning of pancreatic β cells [103]; its deficiency leads to the lowering of insulin sensitivity and increases the risk of CVDs, atherosclerosis, T2DM, and hypertension. Vitamin D3 has been shown in clinical studies to substantially improve sensitivity to insulin and HbA1c. Deficiency of vitamin D3 is associated with T1DM and T2DM [101]. On the other hand, Millen et al. in a large prospective study conducted on over 1300 Americans, found no correlation between dietary vitamin D intake and retinopathy [78]. NO production and maintenance of the integrity of the vascular system are determined by B group vitamins [104]. Pyridoxamine, which is a form of vitamin B6, has the ability to inhibit the later stages of glycation reactions that lead to the formation of advanced glycation end products (AGEs). As a result, vitamin B6 can help protect against the early death of pericyte cells by preserving the viability of capillaries. Pyridoxamine also hinders the formation of acellular strands in the diabetic retina, helping to maintain the presence of microvascular cells [105]. Low concentrations of folic acid (vitamin B9) and cobalamine (vitamin B12) increase the risk of homocysteine vascular damage [106]. Biotin (vitamin B7) supplementation enhances glucose uptake in skeletal muscle cells and increases glucose disposal, thus having a positive effect on glucose management. Potential in improving lipid metabolism has also been found [107].

Zinc is found in spinach, beans, pumpkin, almonds, and oysters, among other products. Zinc is a critical component necessary for cell division, DNA synthesis, immune function, and metabolism of carbohydrates and proteins. Progression of several chronic pathological conditions, such as diabetes, diabetic microvascular complications, and DR metabolic syndrome is associated with zinc deficiency [108,109,110]. The exacerbated duration of diabetes, elevated HbA1c, hypertension, and microcirculatory complications are all correlated with low serum levels of zinc. Serum zinc levels drop gradually with DR duration and severity [110]. Brazil nuts, beans, broccoli, cabbage, spinach, fish, and whole grains are rich in selenium [67]. Selenium prevents some risks associated with DR by downregulating VEGF production [67]. Glutathione peroxidase is an essential antioxidant enzyme responsible for breaking down reactive oxygen species (ROS). It is classified as an enzyme that relies on selenium for its proper functioning [67]. Iron is found in pumpkin, squash, green leafy vegetables, peas, whole grains, lentils, chickpeas, tofu, almonds, and chicken liver [67]. PDR pathogenesis is multifactorial, and iron deficiency anemia may be one of them [111]. Seafood, almonds, and seeds (chia, sesame, and flax) are rich in manganese [67]. Manganese superoxide dismutase overexpression exhibits protective properties toward mitochondrially encoded genes and inhibits mtDNA damage, which possibly plays a role in preventing the mechanism of DR pathogenesis [112,113]. Normal copper levels also play an important role in preventing DR. The insufficient presence of this enzyme leads to disruptions in glucose metabolism, elevated cholesterol levels, weakened antioxidant defense mechanisms, heightened glycation, peroxidation, and nitration caused by diabetes [67]. Copper can be obtained from various food sources, including mushrooms, beans, green leafy vegetables, goat cheese, avocado, seeds (such as chia, sesame, and flax), nuts, and seafood [67].

## 6. DR and the Possible Protective Mechanism of MD

Hyperglycemia and the duration of diabetes mellitus have been found to be the factors most strongly influencing the development and progression of DR [52,114,115]. Hyperglycemia may influence the development of retinopathy through a variety of ways: non-enzymatic protein glycation, protein kinase C activation, polyol pathway, hexosamine pathway activation, accumulation of reactive oxygen species (ROS), and induction of hypoxia-induced factor [52]. Phytochemicals in the MD, such as epigallocatechin-3 glallan, eriodictyol, luteolin, hesperidin, curcumin, and baicalein, have anti-inflammatory effects in the retina by reducing inflammatory cytokines (e.g., vascular endothelial growth factor, IL-1, IL-6, IL-8, and TNF-α) [53,116,117,118]. Therefore, the MD is beneficial in preventing DR through its effects on glycemic control and lipid profiles.

### 6.1. Non-Enzymatic Glycation of Proteins

Non-enzymatic glycation of proteins causes accelerated accumulation of advanced glycation end products (AGEs). AGEs are responsible for the loss of pericytes in the retinal vessels. They lead to the development of inflammation, oxidative stress, and activation of the vascular endothelial growth factor (VEGF) [52,54,119]. VEGF is responsible for pathological retinal neovascularization [52,120]. The highest amounts of AGEs were found in beef [53]. The MD limits the supply of AGE [121,122]. The presence of polyphenols in fruits and vegetables may contribute to their potential protective effect.

Polyphenols improve glucose homeostasis and insulin resistance, and they also reduce inflammation [123]. Vitamins C and E (carotenoids) reduce neovascularization, improve retinal blood flow, and scavenge free radicals [124]. Vitamins C and E have been shown to inhibit VEGF production in animals and reduce AGE accumulation. Taurine present in seafood, shellfish, chicken meat, fish (salmon, tuna, sardines), dairy products, and oatmeal alleviates DR by improving the condition of the retinal vessels and reducing VEGF induction [67,105]. Vitamin B6 (pyridoxamine) inhibits late stages of glycation reactions which result in AGE formation. The potential protective effect of fruits and vegetables may be attributed to the presence of polyphenols in these food sources. Acellular strand formation is also inhibited by vitamin B6 in the diabetic retina, thereby maintaining microvascular cellularity [67,125]. Taurine is capable of lowering VEGF levels in retinal homogenates due to the reduction of oxidative stress [67,126]. In diabetes, the deposition of advanced glycation end products (AGEs) hinders the diffusion of nutrients from the vasculature and creates a hypoxic environment for retinal pigment epithelium and photoreceptors. Treatment with taurine has been found to reduce AGE formation induced by a high-fructose diet. Taurine treatment attenuates the induction of glial fibrillary acid protein (GFAP), a marker of gliosis and apoptosis, in retinal glial cells in diabetes. Furthermore, taurine significantly decreases retinal carbonyl dienes [126].

### 6.2. Reactive Oxygen Intermediates

Oxidative stress is also increased by chronic hyperglycemia. Oxidative phosphorylation of glucose and glucose autoxidation produce free radicals, such as superoxide anions, as byproducts. The elevated glucose levels lead to an increase in their production, resulting in oxidative stress. Oxidative stress, in turn, lowers nitric oxide (NO) levels, enhances leukocyte adhesion to the endothelium, impairs the barrier function of endothelial cells (ECs), damages cellular proteins, and activates protein kinase C (PKC) by promoting diacylglycerol formation. Mitochondrial DNA (mtDNA) and cellular proteins may be damaged by free radicals [64,67]. α-Lipoic acid is a biological antioxidant of reactive oxygen species (ROS) [127]. It inhibits the development of DR by preventing the accumulation of oxidatively modified DNA. Similarly, PUFA metabolites show anti-inflammatory activity by suppressing interleukin (IL)-6, tumor necrosis factor (TNF)-α, VEGF, and ROS, as well as restoring antioxidant homeostasis [67,128]. Bioflavonoids exhibit antioxidant properties by directly scavenging free radicals, inhibiting enzymes responsible for the production of superoxides, and chelation of trace elements that strengthen ROS [67]. Vitamin B9 (folic acid), present in green leafy vegetables and broccoli, and vitamin B12 (cobalamin) found, among others, in crustaceans and fish, reduce the risk of damage to the blood vessels of the retina caused by homocysteine [67]. Homocysteine, incorporated with proteins by a disulfide or amide linkage, may cause severe oxidative stress and inflammation [67].

### 6.3. Protein Kinase C Activation

Pathological activation of protein kinase C (PKC) may result from PKC hyperglycemia. Protein kinase C (PKC) is an enzyme that is frequently associated with vascular damage, as it can lead to increased vascular permeability, dysregulation of nitric oxide (NO), enhanced adhesion of leukocytes to vessel walls, and disturbances in blood flow. Importantly, these effects of PKC occur independently of the involvement of the aldose reductase pathway. MAPK or NF-κB pathways may be influenced by protein kinase C activation [67,129].

Vitamin C may also reduce the activation of protein kinase C, prevent pericyte apoptosis, and reduce oxidative stress in the retinal pigment epithelium [52].

### 6.4. Polyol Pathway

Increased levels of glucose lead to heightened activation of the aldose reductase pathway, also referred to as the polyol pathway. Consequently, there is a decline in intracellular levels of nicotinamide adenine dinucleotide phosphate (NADP), resulting in reduced production of nitric oxide (NO) within endothelial cells (ECs). Additionally, this pathway induces chronic galactosemia, which leads to alterations in the basement membrane of blood vessels, loss of pericytes, formation of microaneurysms, and the presence of cell-free capillaries. Excessive amounts of galactose compete with glucose for glucose transporters (GLUT), thus limiting the entry of glucose into retinal cells and reducing cellular energy metabolism that requires glucose [67,128]. It has been reported that ethanol is associated with a decrease in the level of glutathione, an important antioxidant [52]. Glutathione may also increase lipid peroxidation and the production of free radicals [52]. 

## 7. The Role of MD in the Treatment and Prevention of Obesity, and BMI as a Factor in the Development of DR

According to the World Health Organization (WHO), obesity is defined as “abnormal or excessive fat accumulation in adipose tissue to the extent that health may be impaired”. The World Obesity Federation (WOF) recognizes obesity as a chronic, recurring, and progressive disease [129]. In the latest edition of the International Classification of Diseases (ICD-11) by the WHO, the stigmatizing diagnosis of “obesity due to excess calories” has been discontinued. Obesity is also diagnosed when the body mass index (BMI) exceeds 30 kg/m^2^ [130]. Previous research has demonstrated a positive correlation between BMI and the risk of dyslipidemia. Around 60–70% of obese individuals are affected by dyslipidemia, while the prevalence in overweight individuals is approximately 50–60% [131,132]. Obesity is a significant social problem. It predisposes to the development of carbohydrate metabolism, hypertension, and ischemic stroke, which may increase the risk of developing retinopathy in people during prediabetes as well as those diagnosed with diabetes [133,134,135]. However, the findings from various studies investigating the relationship between obesity and DR have been inconclusive. Kostev et al. conducted an analysis of databases in Great Britain and found no significant association between body mass index (BMI) and the presence of diabetic retinopathy in patients with T2DM [136]. In other studies, the authors observed a significant deterioration of HbA1c and a significant increase in cholesterol and hypertension with an increase in BMI. The correlation between BMI and triglycerides was not significant. Therefore, the authors propose that the correlation between BMI and factors such as HbA1c, cholesterol, and hypertension seems to be linked to the advancement of diabetic retinopathy (DR) in individuals with type 2 diabetes. This relationship may potentially serve as a predictive indicator for the emergence of this significant contributor to visual impairment in developed nations [137]. In the study of Kastelan et al., 176 patients with type 1 diabetes with a mean age of 40.03 ± 14.66 years were examined. A notable decline in HbA1c levels, elevation in total cholesterol levels, as well as increased systolic and diastolic blood pressure were observed alongside the advancement of retinopathy. The progression of diabetic retinopathy was found to be linked to the duration of diabetes, HbA1c levels, hypertension, total cholesterol levels, and the presence of diabetic nephropathy. Among patients without nephropathy, statistical analysis revealed a significant correlation between higher BMI and the progression of retinopathy. The study findings revealed a positive association between BMI and a notable worsening of HbA1c levels, an increase in cholesterol and triglyceride levels, as well as the presence of hypertension. Based on their observations, the researchers propose that BMI, in conjunction with HbA1c, cholesterol, and hypertension, may play a role in the progression of diabetic retinopathy in individuals with type 1 diabetes who do not suffer from nephropathy [138].

Price et al. conducted a study indicating that obesity (BMI > 30 kg/m^2^) is the main risk factor for the development of DR in people with T1DM [139]. Similar findings were reported by Grauslund et al. [140]. Forga et al., in a 10-year study of 989 patients with T1DM, found that baseline BMI did not show a significant association with the development of DR. However, the risk increased with the time of observation [141]. A study conducted in Sweden showed that in T1DM and T2DM patients, the occurrence of DR was correlated with a high BMI [142]. It was also shown that abdominal obesity measured by waist circumference (WC) and waist-to-hip ratio (WHR) was positively associated with DR in patients with T1DM and T2DM [143,144]. Waist circumference (WC) was higher in patients with DR than in patients without DR. Similarly, the waist-to-hip ratio (WHR) was higher in patients with DR than in patients without DR [144]. However, no correlation was found between abdominal obesity and the severity of diabetic retinopathy [144]. 

The development of DR in obese people with diabetes is also explained by elevated leptin levels [145]. The authors showed that in patients with proliferative diabetic retinopathy (PDR), the average vitreous level of leptin (37.4 ng/mL) was significantly higher than that in patients with proliferative vitreoretinopathy (PVR) (<1.0 ng/mL, *p* < 0.05). The levels of vitreous leptin in patients with proliferative vitreoretinopathy (PVR) or macular disease, with or without diabetes, did not show significant differences compared with the control group consisting of individuals with retinal detachment alone. However, the findings indicate that the level of leptin in vitreous samples is elevated in proliferative diabetic retinopathy (PDR). Based on these results, the authors propose that leptin may have an active role in the development and progression of PDR [145]. Leptin is a 167–amino acid protein transcribed from the o b gene, which was originally cloned from the adipose tissue of mice and human subjects [146]. The leptin gene is expressed, among others, in adipose tissue, and the level of leptin in plasma positively correlates with the content of adipose tissue. Since leptin is angiogenic in vitro and induces neovascularization in vivo [146], obesity may lead to DR through elevated leptin levels [145]. 

In other studies, authors demonstrated a strong correlation between adiponectin levels in the bloodstream and the presence and advancement of diabetic retinopathy (DR). Furthermore, the levels of adiponectin in the aqueous humor have shown a similar correlation. It has been suggested that serum adiponectin may serve as a more reliable indicator for estimating the levels of intraocular cytokines, including both intraocular adiponectin and vascular endothelial growth factor (VEGF), in clinical settings for patients with DR, compared with serum VEGF alone [147]. Elhayany et al. showed that overweight (average BMI of 31.4) patients with diabetes who followed a MD lost an average of 7.4 kg of body weight after 12 months [148]. Similar results were obtained in other clinical trials [149,150]. In obese adults, sustained weight loss (up to 12 months) was shown to be greater in the MD compared with a low-fat diet (mean range: −4.1 to −10.1 kg vs. 2.9 to −5.0 kg) but similar to the low-carbohydrate diet (4.1 to −10.1 kg vs. −4.7 to −7.7 kg) [149,150]. In contrast, Esposito et al. showed that after 1 year of following a MD and a low-fat diet, the level of HbA1c in 215 patients with type 2 DM was lower (0.4% to 0.6%) in people following the MD compared with those following a low-fat diet [151]. Similar results were reported by Elhayany et al. [148]. In a 12-month study, they found a significant difference in HbA1c reduction in people following a MD compared with a low-fat diet [148]. Estruch et al. found that individuals with diabetes and a high risk of cardiovascular diseases who followed the MD along with nuts for 3 months experienced an average decrease in systolic blood pressure of −7.1 mm Hg and a reduced HDL-C ratio of −0.26 compared with those following a low-fat diet [152]. They also observed a decrease in fasting plasma glucose levels by −30.58 mmol/L [152].

The presented data suggest that adopting a MD, which promotes weight reduction and decreases body fat, has a beneficial impact on glucose, lipid, and blood pressure levels in individuals with diabetes. Consequently, it may contribute to a reduced risk of developing diabetic retinopathy (DR). Given the significance of this finding, further research is required.

## 8. Conclusions

In 2003, the WHO recognized the MD as an exemplary healthy diet for both children and adults. Some researchers believe that due to the high-fat content, this diet should not be recommended to the obese [153]. However, evidence suggests that the MD does not result in weight gain or an increase in waist circumference. The EPIC research demonstrated that subjects with high adherence to the MD lost 0.16 kg and were 10% less at risk of becoming overweight or obese than participants with lower adherence [154]. The results of the CARDIA study also showed a reduction in waist circumference among participants following the MD [155]. Due to the wide range of natural products, it exhibits antioxidant, chemopreventive, and anti-inflammatory effects. It reduces the level of triglycerides and cholesterol, as well as postprandial glycemia [19]. It is an important factor in reducing the risk of developing diabetes and is suspected to play a significant role in the development of diabetic retinopathy; however, research in this area is still limited. Given that retinopathy affects a third of people with diabetes, further prospective studies are needed. It is also important to promote MD as a healthy dietary habit so that it is not limited to local popularity. Since diabetes and its complications are common throughout the world, we ought to do everything in our power to minimize its effects.

## Data Availability

Not applicable.

## References

[1] International Diabetes Federation Diabetes Facts and Figures. https://idf.org/aboutdiabetes/what-is-diabetes/facts-figures.html.

[2] Williams R., Colagiuri S., Chan J., Gregg E.W., Ke C., Lim L.-L., Yang X. (2019). IDF Diabetes Atlas 2019; International Diabetes Foundation. https://www.diabetesatlas.org/upload/resources/material/20200302_133351_IDFATLAS9e-final-web.pdf.

[3] Mayer-Davis E.J., Lawrence J.M., Dabelea D., Divers J., Isom S., Dolan L., Imperatore G., Lindre B., Marcovina S., Pettitt D.J. (2017). Incidence Trends of Type 1 and Type 2 Diabetes among Youths, 2002–2012. N. Engl. J. Med..

[4] Zorena K., Myśliwska J., Myśliwiec M., Rybarczyk-Kapturska K., Malinowska E., Wiśniewski P., Raczyńska K. (2010). Association between vascular endothelial growth factor and hypertension in children and adolescents type I diabetes mellitus. J. Hum. Hypertens..

[5] Romero-Aroca P., Navarro-Gil R., Valls-Mateu A., Sagarra-Alamo R., Moreno-Ribas A., Soler N. (2017). Differences in incidence of diabetic retinopathy between type 1 and 2 diabetes mellitus: A nine-year follow-up study. Br. J. Ophthalmol..

[6] Calella P., Vitucci D., Zanfardino A., Cozzolino F., Terracciano A., Zanfardino F., Rollato S., Piscopo A., Gallè F., Mancini A. (2023). Lifestyle and physical fitness in adolescents with type 1 diabetes and obesity. Heliyon.

[7] Bryl A., Mrugacz M., Falkowski M., Zorena K. (2022). The Effect of Diet and Lifestyle on the Course of Diabetic Retinopathy-A Review of the Literature. Nutrients.

[8] Keys A., Menotti A., Karvonen M.J., Aravanis C., Blackburn H., Buzina R., Djordjevic B.S., Dontas A.S., Fidanza F., Keys M.H. (1986). The diet and 15-year death rate in the seven countries study. Am. J. Epidemiol..

[9] Pitsavos C., Panagiotakos D.B., Tzima N., Chrysohoou C., Economou M., Zampelas A., Stefanadis C. (2005). Adherence to the Mediterranean diet is associated with total antioxidant capacity in healthy adults: The ATTICA study. Am. J. Clin. Nutr..

[10] Prokofyeva E., Zrenner E. (2012). Epidemiology of major eye diseases leading to blindness in Europe: A literature review. Ophthalmic Res..

[11] Resnikoff S., Pascolini D., Etya’ale D., Kocur I., Pararajasegaram R., Pokharel G.P., Mariotti S.P. (2004). Global data on visual impairment in the year 2002. Bull. World Health Organ..

[12] Mrugacz M., Bryl A., Zorena K. (2021). Retinal Vascular Endothelial Cell Dysfunction and Neuroretinal Degeneration in Diabetic Patients. J. Clin. Med..

[13] Saeedi P., Petersohn I., Salpea P., Malanda B., Karuranga S., Unwin N., Colagiuri S., Guariguata L., Motala A.A., Ogurtsova K. (2019). IDF Diabetes Atlas Committee. Global and regional diabetes prevalence estimates for 2019 and projections for 2030 and 2045: Results from the International Diabetes Federation Diabetes Atlas, 9(th) edition. Diabetes Res. Clin. Pract..

[14] Guidelines for the Management of Diabetic Retinopathy Guidelines for Diabetic Retinopathy. https://www.optometry.org.au/wp-content/uploads/Professional_support/Guidelines/nhmrc_diabetic_guidelines.pdf.

[15] Pesin N., Mandelcorn E.D., Felfeli T., Ogilvie R.I., Brent M.H. (2017). The role of occult hypertension in retinal vein occlusions and diabetic retinopathy. Can. J. Ophthalmol..

[16] From the American Association of Neurological Surgeons (AANS), American Society of Neuroradiology (ASNR), Cardiovascular and Interventional Radiology Society of Europe (CIRSE), Canadian Interventional Radiology Association (CIRA), Congress of Neurological Surgeons (CNS), European Society of Minimally Invasive Neurological Therapy (ESMINT), European Society of Neuroradiology (ESNR), European Stroke Organization (ESO), Society for Cardiovascular Angiography and Interventions (SCAI), Society of Interventional Radiology (SIR) (2018). Multisociety consensus quality improvement revised consensus statement for endovascular therapy of acute ischemic stroke. Int. J. Stroke.

[17] Mozetic V., Freitas C.G., Riera R. (2017). Statins and fibrates for diabetic retinopathy: Protocol for a systematic review. JMIR Res. Protoc..

[18] Ibsen D.B., Overvad K., Laursen A.S.D., Halkjær J., Tjønneland A., Kilpeläinen T.O., Parner E.T., Jakobsen M.U. (2021). Changes in intake of dairy product subgroups and risk of type 2 diabetes: Modelling specified food substitutions in the Danish Diet, Cancer and Health cohort. Eur. J. Nutr..

[19] Shah J., Cheong Z.Y., Tan B., Wong D., Liu X., Chua J. (2022). Dietary Intake and Diabetic Retinopathy: A Systematic Review of the Literature. Nutrients.

[20] Mańkiewicz-Żurawska I., Jarosz-Chobot P. (2019). Nutrition of children and adolescents with type 1 diabetes in the recommendations of the Mediterranean diet. Pediatr. Endocrinol. Diabetes Metab..

[21] Li T.Y., Brennan A.M., Wedick N.M., Mantzoros C., Rifai N., Hu F.B. (2009). Regular consumption of nuts is associated with a lower risk of cardiovascular disease in women with type 2 diabetes. J. Nutr..

[22] Prabhakaran D., Khandelwal S., Martínez-González M.A., Tong T.Y., Forouhi N.G., Trichopoulou A., Mozaffarian D., de Lorgeril M. (2014). Definitions and Potential Health Benefits of the Mediterranean Diet: Views from Experts around the World. BMC Med..

[23] García-González D., Aparicio-Ruiz R., Aparicio R. (2008). Virgin olive oil—Chemical implications on quality and health. Eur. J. Lipid Sci. Technol..

[24] Kłosiewicz-Latoszek L. (2009). Dietary guidelines in prevention of chronic diseases. Probl. Hig. Epidemiol..

[25] Martinez-Gonzalez M.A., de la Fuente-Arrillaga C., Nunez-Cordoba J.M., Basterra-Gortari F.J., Beunza J.J., Vazquez Z., Benito S., Tortosa A., Bes-Rastrollo M. (2008). Adherence to mediterranean diet and risk of developing diabetes: Prospective cohort study. BMJ.

[26] European Union Regolamento (CEE). http://data.europa.eu/eli/reg/1991/2568/2015-01-01.

[27] Trichopoulou A., Critselis E. (2004). Mediterranean Diet and Longevity. Eur. J. Cancer Prev..

[28] Georgoulis M., Kontogianni M.D., Yiannakouris N. (2014). Mediterranean diet and diabetes: Prevention and treatment. Nutrients.

[29] Tuttolomondo A., Simonetta I., Daidone M., Mogavero A., Ortello A., Pinto A. (2019). Metabolic and Vascular Effect of the Mediterranean Diet. Int. J. Mol. Sci..

[30] Evert A.B., Dennison M., Gardner C.D., Garvey W.T., Lau K.H.K., MacLeod J., Mitri J., Pereira R.F., Rawlings K., Robinson S. (2019). Nutrition therapy for adults with diabetes or prediabetes: A consensus report. Diabetes Care.

[31] Cadario F., Prodam F., Pasqualicchio S., Bellone S., Bonsignori I., Demarchi I., Monzani A., Bona G. (2012). Lipid profile and nutritional intake in children and adolescents with Type 1 diabetes improve after a structured dietician training to a Mediterranean-style diet. J. Endocrinol. Investig..

[32] Zhong V.W., Lamichhane A.P., Crandell J.L., Couch S.C., Liese A.D., The N.S., Tzeel B.A., Dabelea D., Lawrence J.M., Marcovina S.M. (2016). Association of adherence to a Mediterranean diet with glycemic control and cardiovascular risk factors in youth with type I diabetes: The SEARCH Nutrition Ancillary Study. Eur. J. Clin. Nutr..

[33] Dominguez-Riscart J., Buero-Fernandez N., Garcia-Zarzuela A., Morales-Perez C., Garcia-Ojanguren A., Lechuga-Sancho A.M. (2022). Adherence to Mediterranean Diet Is Associated with Better Glycemic Control in Children with Type 1 Diabetes: A Cross-Sectional Study. Front. Nutr..

[34] Schoenaker D.A.J.M., Toeller M., Chaturvedi N., Fuller J.H., Soedamah-Muthu S.S., EURODIAB Prospective Complications Study Group (2012). Dietary saturated fat and fibre and risk of cardiovascular disease and all-cause mortality among type 1 diabetic patients: The EURODIAB Prospective Complications Study. Diabetologia.

[35] Antoniotti V., Spadaccini D., Ricotti R., Carrera D., Savastio S., Goncalves Correia F.P., Caputo M., Pozzi E., Bellone S., Rabbone I. (2022). Adherence to the Mediterranean Diet Is Associated with Better Metabolic Features in Youths with Type 1 Diabetes. Nutrients.

[36] García Cabrera S., Herrera Fernández N., Rodríguez Hernández C., Nissensohn M., Román-Viñas B., Serra-Majem L. (2015). KIDMED Test; prevalence of low adherence to the Mediterranean Diet in children and young; A systematic review. Nutr. Hosp..

[37] Panagiotakos D.B., Tzima N., Pitsavos C., Chrysohoou C., Zampelas A., Toussoulis D., Stefanadis C. (2007). The association between adherence to the Mediterraneandiet and fasting indices of glucose homoeostasis: The Attica study. J. Am. Coll. Nutr..

[38] Ortega E., Franch J., Castell C., Goday A., Ribas-Barba L., Soriguer F., Vendrell J., Casamitjana R., Bosch-Comas A., Bordiu E. (2013). Mediterranean diet adherence in individuals with prediabetes and unknown diabetes: The di@bet.es study. Ann. Nutr. Metab..

[39] Mozaffarian D., Marfisi R., Levantesi G., Silletta M.G., Tavazzi L., Tognoni G., Valagussa F., Marchioli R. (2007). Incidence of new-onset diabetes and impaired fasting glucose in patients with recent myocardial infarction and the effect of clinical and lifestyle risk factors. Lancet.

[40] De Koning L., Chiuve S.E., Fung T.T., Willett W.C., Rimm E.B., Hu F.B. (2011). Diet-quality scores and the risk of type 2 diabetes in men. Diabetes Care.

[41] Abiemo E.E., Alonso A., Nettleton J.A., Steffen L.M., Bertoni A.G., Jain A., Lutsey P.L. (2013). Relationships of the mediterranean dietary pattern with insulin resistance and diabetes incidence in the multi-ethnic study of atherosclerosis (MESA). Br. J. Nutr..

[42] Rossi M., Turati F., Lagiou P., Trichopoulos D., Augustin L.S., La Vecchia C., Trichopoulou A. (2013). Mediterranean diet and glycaemic load in relation to incidence of type 2 diabetes: Results from the greek cohort of the population-based european prospective investigation into cancer and nutrition (EPIC). Diabetologia.

[43] Ajala O., English P., Pinkney J. (2013). Systematic review and meta-analysis of different dietary approaches to the management of type 2 diabetes. Am. J. Clin. Nutr..

[44] Ciccarone E., di Castelnuovo A., Salcuni M., Siani A., Giacco A., Donati M.B., de Gaetano G., Capani F., Iacoviello L., Gendiabe I. (2003). A high-score mediterranean dietary pattern is associated with a reduced risk of peripheral arterial disease in italian patients with type 2 diabetes. J. Thromb. Haemost..

[45] De Lorgeril M., Salen P., Martin J.L., Monjaud I., Delaye J., Mamelle N. (1999). Mediterranean diet, traditional risk factors, and the rate of cardiovascular complications after myocardial infarction: Final report of the lyon diet heart study. Circulation.

[46] Barzi F., Woodward M., Marfisi R.M., Tavazzi L., Valagussa F., Marchioli R., Investigators G.I.-P. (2003). Mediterranean diet and all-causes mortality after myocardial infarction: Results from the gissi-prevenzione trial. Eur. J. Clin. Nutr..

[47] Karamanos B., Thanopoulou A., Anastasiou E., Assaad-Khalil S., Albache N., Bachaoui M., Slama C.B., El Ghomari H., Jotic A., Lalic N. (2013). Relation of the mediterranean diet with the incidence of gestational diabetes. Eur. J. Clin. Nutr..

[48] Valero-Vello M., Peris-Martínez C., García-Medina J.J., Sanz-González S.M., Ramírez A.I., Fernández-Albarral J.A., Galarreta-Mira D., Zanón-Moreno V., Casaroli-Marano R.P., Pinazo-Duran M.D. (2021). Searching for the Antioxidant, Anti-Inflammatory, and Neuroprotective Potential of Natural Food and Nutritional Supplements for Ocular Health in the Mediterranean Population. Foods.

[49] Robles-Rivera R.R., Castellanos-González J.A., Olvera-Montaño C., Flores-Martin R.A., López-Contreras A.K., Arevalo-Simental D.E., Cardona-Muñoz E.G., Roman-Pintos L.M., Rodríguez-Carrizalez A.D. (2020). Adjuvant Therapies in Diabetic Retinopathy as an Early Approach to Delay Its Progression: The Importance of Oxidative Stress and Inflammation. Oxid. Med. Cell. Longev..

[50] Chiosi F., Rinaldi M., Campagna G., Manzi G., De Angelis V., Calabrò F., D’Andrea L., Tranfa F., Costagliola C. (2022). Effect of a Fixed Combination of Curcumin, Artemisia, Bromelain, and Black Pepper Oral Administration on Optical Coherence Tomography Angiography Indices in Patients with Diabetic Macular Edema. Nutrients.

[51] Safi S.Z., Qvist R., Kumar S., Batumalaie K., Ismail I.S. (2014). Molecular mechanisms of diabetic retinopathy, general preventive strategies, and novel therapeutic targets. BioMed Res. Int..

[52] Dow C., Mancini F., Rajaobelina K., Boutron-Ruault M.C., Balkau B., Bonnet F., Fagherazzi G. (2018). Diet and risk of diabetic retinopathy: A systematic review. Eur. J. Epidemiol..

[53] Calder P.C., Ahluwalia N., Brouns F., Buetler T., Clement K., Cunningham K., Esposito K., Jönsson L.S., Kolb H., Lan-sink M. (2011). Dietary factors and low-grade inflammation in relation to overweight and obesity. Br. J. Nutr..

[54] Díaz-López A., Babio N., Martínez-González M.A., Corella D., Amor A.J., Fitó M., Estruch R., Arós F., Gómez-Gracia E., Fiol M. (2015). PREDIMED Study Investigators. Mediterranean Diet, Retinopathy, Nephropathy, and Microvascular Diabetes Complications: A Post Hoc Analysis of a Randomized Trial. Diabetes Care.

[55] Ghaemi F., Firouzabadi F.D., Moosaie F., Shadnoush M., Poopak A., Kermanchi J., Abhari S.M.F., Forouzanfar R., Mansournia M.A., Khosravi A. (2021). Effects of a Mediterranean diet on the development of diabetic complications: A longitudinal study from the nationwide diabetes report of the National Program for Prevention and Control of Diabetes (NPPCD 2016-2020). Maturitas.

[56] Bryl A., Falkowski M., Zorena K., Mrugacz M. (2022). The Role of Resveratrol in Eye Diseases—A Review of the Literature. Nutrients.

[57] Galiniak S., Aebisher D., Bartusik-Aebisher D. (2019). Health benefits of resveratrol administration. Acta Biochim. Pol..

[58] Kim Y.H., Kim Y.S., Roh G.S., Choi W.S., Cho G.J. (2012). Resveratrol blocks diabetes-induced early vascular lesions and vascular endothelial growth factor induction in mouse retinas. Acta Ophthalmol..

[59] Losso J.N., Truax R.E., Richard G. (2010). Trans-resveratrol inhibits hyperglycemia-induced inflammation and connexin downregulation in retinal pigment epithelial cells. J. Agric. Food Chem..

[60] Mahoney S.E., Loprinzi P.D. (2014). Influence of flavonoid-rich fruit and vegetable intake on diabetic retinopathy and diabetes-related biomarkers. J. Diabetes Complicat..

[61] Tanaka S., Yoshimura Y., Kawasaki R., Kamada C., Tanaka S., Horikawa C., Ohashi Y., Araki A., Ito H., Akanuma Y. (2013). Fruit intake and incident diabetic retinopathy with type 2 diabetes. Epidemiology.

[62] Lyons M.M., Yu C., Toma R.B., Cho S.Y., Reiboldt W., Lee J., van Breemen R.B. (2003). Resveratrol in raw and baked blueberries and bilberries. J. Agric. Food Chem..

[63] Wang Y., Catane F., Yang Y., Roderick R., Van Breemen R.B. (2002). An LC-MS method for analysing total resveratrol in grape juice, cranberry juice and in wine. J. Agric. Food Chem..

[64] Packer L., Witt E., Tritchler H. (1995). Alpha-lipoic acid as a biological antioxidant. Free Radic. Biol. Med..

[65] Kowluru R.A., Odenbach S. (2004). Effect of long term administration of alpha lipoic acid on retinal capillary cell death and the development of retinopathy in diabetic rats. Diabetes.

[66] Lin J., Bierhaus A., Bugert P., Dietrich N., Feng Y., Vom Hagen F., Nawroth P., Brownlee M., Hammes H.P. (2006). Effect of R-(+)-alpha-lipoic acid on experimental diabetic retinopathy. Diabetologia.

[67] Sharma Y., Saxena S., Mishra A., Saxena A., Natu S.M. (2017). Nutrition for diabetic retinopathy: Plummeting the inevitable threat of diabetic vision loss. Eur. J. Nutr..

[68] Buttriss J.L., Stokes C.S. (2008). Dietary fibre and health: An overview. Br. Nutr. Found. Nutr. Bull..

[69] Williams J.H., Patel P., Jelfs R., Carter R.D., Awdry P., Bron A., Mann J.I., Hockaday T.D.R. (1985). Polyunsaturated fatty acids and diabetic retinopathy. Br. J. Ophthalmol..

[70] Bienkiewicz M., Bator E., Bronkowska M. (2015). Dietary fiber and its importance in health promotion. Probl. Hig. Epidemiol..

[71] Fujii H., Iwase M., Okhuma T., Ogata-Kaizu S., Ide H., Kikuchi Y., Idewaki Y., Joudai T., Hirakawa Y., Uchida K. (2013). Impact of dietary fiber intake on glycemic control, cardiovascular risk factors and chronic kidney disease in Japanese patients with type 2 diabetes mellitus: The Fukuoka Diabetes Registry. Nutr. J..

[72] Ganesan S., Raman R., Kulothungan V., Sharma T. (2012). Influence of dietary-fibre intake on diabetes and diabetic retinopathy: SankaraNethralaya-Diabetic Retinopathy Epidemiology and Molecular Genetic Study (report 26). Clin. Exp. Ophthalmol..

[73] Sasaki M., Kawasaki R., Rogers S., Man R.E.K., Itakura K., Xie J., Flood V., Tsubota K., Lamoureux E., Wang J.J. (2015). The Associations of Dietary Intake of Polyunsaturated fatty acids with diabetic retinopathy in well-controlled diabetes. Investig. Ophthalmol. Vis. Sci..

[74] Alcubierre N., Navarrete-Muñoz E.M., Rubinat E., Falguera M., Valls J., Traveset A., Vilanova M.-B., Marsal J.R., Hernandez M., Granado-Casas M. (2016). Association of low oleic acid intake with diabetic retinopathy in type 2 diabetic patients: A casecontrol study. Nutr. Metab..

[75] Sala-Vila A., Díaz-López A., Valls-Pedret C., Cofán M., García-Layana A., Lamuela-Raventós R.M., Castañer O., Zanon-Moreno V., Martinez-Gonzalez M.A., Toledo E. (2016). Dietary Marine omega-3 fatty acids and incident sight-threatening retinopathy in middleaged and older individuals with type 2 diabetes: Prospective investigation from the PREDIMED trial. JAMA Ophthalmol..

[76] Yanai R., Mulki L., Hasegawa E., Takeuchi K., Sweigard H., Suzuki J., Gaissert P., Vavvas D.G., Sonoda K.H., Rothe M. (2014). Cytochrome P450-generated metabolites derived from ω-3 fatty acids attenuate neovascularization. Proc. Natl. Acad. Sci. USA.

[77] Rosenberg K. (2017). Omega-3 Fatty Acid Intake Lowers Risk of Diabetic Retinopathy. Am. J. Nurs..

[78] Millen A.E., Sahli M.W., Nie J., LaMonte M.J., Lutsey P.L., Klein B.E.K., Mares J.A., Meyers K.J., Andrews C.A., Klein R. (2016). Adequate vitamin D status is associated with the reduced odds of prevalent diabetic retinopathy in African Americans and Caucasians. Cardiovasc. Diabetol..

[79] Marushka L., Batal M., David W., Schwartz H., Ing A., Fediuk K., Sharp D., Black A., Tikhonov C., Chan H.M. (2017). Association between fish consumption, dietary omega-3 fatty acids and persistent organic pollutants intake, and type 2 diabetes in 18 First Nations in Ontario, Canada. Environ. Res..

[80] Mirmiran P., Hosseinpour-Niazi S., Naderi Z., Bahadoran Z., Sadeghi M., Azizi F. (2012). Association between interaction and ratio of ω-3 and ω-6 polyunsaturated fatty acid and the metabolic syndrome in adults. Nutrition.

[81] Chew E.Y. (2017). Dietary Intake of Omega-3 Fatty Acids from Fish and Risk of Diabetic Retinopathy. JAMA.

[82] Mayor S. (2016). Oily fish intake reduces risk of diabetic retinopathy, study shows. BMJ.

[83] Yee P., Weymouth A.E., Fletcher E.L., Vingrys A.J. (2010). A role for Omega-3 polyunsaturated fatty acid supplements in diabetic neuropathy. Investig. Ophthalmol..

[84] Alsbirk K.E., Seland J.H., Assmus J. (2022). Diabetic retinopathy and visual impairment in a Norwegian diabetic coast population with a high dietary intake of fish oils. An observational study. Acta Ophthalmol..

[85] Meyer B.J. (2011). Are we consuming enough long chain omega-3 polyunsaturated fatty acids for optimal health?. Prostaglandins Leukot. Essent. Fat. Acids.

[86] Kawasaki R., Tanaka S., Tanaka S., Yamamoto T., Sone H., Ohashi Y., Akanuma Y., Yamada N., Yamashita H. (2011). Incidence and progression of diabetic retinopathy in Japanese adults with type 2 diabetes: 8 year follow-up study of the Japan Diabetes Complications Study (JDCS). Japan Diabetes Complications Study Group. Diabetologia.

[87] Horikawa C., Yoshimura Y., Kamada C., Tanaka S., Tanaka S., Hanyu O., Araki A., Ito H., Tanaka A., Ohashi Y. (2014). Dietary sodium intake and incidence of diabetes complications in Japanese patients with type 2 diabetes: Analysis of the Japan Diabetes Complications Study (JDCS). J. Clin. Endocrinol. Metab..

[88] Engelen L., Soedamah-Muthu S.S., Geleijnse J.M., Toeller M., Chaturvedi N., Fuller J.H., Schalkwijk C.G., Stehouwer C.D.A. (2014). Higher dietary salt intake is associated with microalbuminuria, but not with retinopathy in individuals with type 1 diabetes: The EURODIAB Prospective Complications Study. Diabetologia.

[89] Mayer-Davis E.J., Bell R.A., Reboussin B.A., Rushing J., Marshall J.A., Hamman R.F. (1998). Antioxidant nutrient intake and diabetic retinopathy: The San Luis Valley Diabetes Study. Ophthalmology.

[90] Sahli M.W., Mares J.A., Meyers K.J., Klein R., Brady W.E., Klein B.E., Ochs-Balcom H.M., Donahue R.P., Millen A.E. (2016). Dietary intake of lutein and diabetic retinopathy in the atherosclerosis risk in Communities Study (ARIC). Ophthalmic Epidemiol..

[91] Brazionis L., Rowley K., Itsiopoulos C., O’Dea K. (2009). Plasma carotenoids and diabetic retinopathy. Br. J. Nutr..

[92] Garcia-Medina J.J., Pinazo-Duran M.D., Garcia-Medina M., Zanon-Moreno V., Pons-Vazquez S. (2011). A 5-year follow-up of antioxidant supplementation in type 2 diabetic retinopathy. Eur. J. Ophthalmol..

[93] Zhang P.-C., Wu C.-R., Wang Z.-L., Wang L.-Y., Han Y., Sun S.-L., Li Q.-S., Ma L. (2017). Effect of lutein supplementation on visual function in nonproliferative diabetic retinopathy. Asia Pac. J. Clin. Nutr..

[94] Millen A.E., Klein R., Folsom A.R., Stevens J., Palta M., Mares J.A. (2004). Relation between intake of vitamins C and E and risk of diabetic retinopathy in the Atherosclerosis Risk in Communities Study. Am. J. Clin. Nutr..

[95] Thosar S.S., Bielko S.L., Wiggins C.C., Klaunig J.E., Mather K.J., Wallace J.P. (2015). Antioxidant vitamin C prevents decline in endothelial function during sitting. Med. Sci. Monit..

[96] Park S.W., Ghim W., Oh S., Kim Y., Park U.C., Kang J., Yu H.G. (2019). Association of vitreous vitamin C depletion with diabetic macular ischemia in proliferative diabetic retinopathy. PLoS ONE.

[97] Gurreri A., Pazzaglia A., Schiavi C. (2019). Role of statins and ascorbic acid in the natural history of diabetic retinopathy: A new, affordable therapy?. Ophthalmic Surg. Lasers Imaging Retina.

[98] Bursell S.E., Clermont A.C., Aiello L.P., Aiello L.M., Schlossman D.K., Feener E.P., Laffel L.O.R.L., King G.L. (1999). High-dose vitamin E supplementation normalizes retinal blood flow and creatinine clearance in patients with type 1 diabetes. Diabetes Care.

[99] Chatziralli I.P., Theodossiadis G., Dimitriadis P., Charalambidis M., Agorastos A., Migkos Z., Platogiannis N., Moschos M.M., Theodossiadis P., Keryttopoulos P. (2017). The effect of vitamin E on oxidative stress indicated by serum malondialdehyde in insulin-dependent type 2 diabetes mellitus patients with retinopathy. Open Ophthalmol. J..

[100] Stoyanovsky D.A., Goldman R., Darrow R.M., Organisciak D.T., Kagan V.E. (1995). Endogenous ascorbate regenerates vitamin E in the retina directly and in combination with exogenous dihydrolipoic acid. Curr. Eye Res..

[101] Shi C., Wang P., Airen S., Brown C., Liu Z., Townsend J.H., Wang J., Jiang H. (2020). Nutritional and medical food therapies for diabetic retinopathy. Eye Vis..

[102] Long M., Wang C., Liu D. (2017). Glycated hemoglobin A1C and vitamin D and their association with diabetic retinopathy severity. Nutr. Diabetes.

[103] Rashidi B., Hoseini Z., Sahebkar A., Mirzaei H. (2017). Anti-Atherosclerotic Effects of Vitamins D and E in Suppression of Atherogenesis. J. Cell. Physiol..

[104] Smolek M.K., Notaroberto N.F., Jaramillo A.G., Pradillo L.R. (2013). Intervention with vitamins in patients with nonproliferative diabetic retinopathy: A pilot study. Clin. Ophthalmol..

[105] Stitt A., Gardiner T.A., Alderson N.L., Canning P., Frizzell N., Duffy N., Boyle C., Januszewski A.S., Chachich M., Baynes J.W. (2002). The AGE inhibitor pyridoxamine inhibits development of retinopathy in experimental diabetes. Diabetes.

[106] Satyanarayana A., Balakrishna N., Pitla S., Reddy P.Y., Mudili S., Lopamudra P., Suryanarayana P., Viswanath K., Ayyagari R., Reddy G.B. (2011). Status of B vitamins and homocysteine in diabetic retinopathy: Association with vitamin B12 deficiency and hyperhomocysteinemia. PLoS ONE.

[107] Singer G.M., Geohas J. (2006). The effect of chromium picolinate and biotin supplementation on glycemic controlin poorly controlled patients with type 2 diabetes mellitus: A placebocontrolled, double-blinded, randomized trial. Diabetes Technol. Ther..

[108] Miao X., Sun W., Miao L., Fu Y., Wang Y., Su G., Liu Q. (2013). Zinc and diabetic retinopathy. J. Diabetes Res..

[109] Prasad A.S. (2013). Discovery of human zinc deficiency: Its impact on human health and disease. Adv. Nutr..

[110] Luo Y.Y., Zhao J., Han X.Y., Zhou X.H., Wu J., Ji L.N. (2015). Relationship between serum zinc level and microvascular complications in patients with type 2 diabetes. Chin. Med. J..

[111] Shorb S.R. (1985). Anemia and diabetic retinopathy. Am. J. Ophthalmol..

[112] Kanwar M., Chan P.S., Kern T.S., Kowluru R.A. (2007). Oxidative damage in the retinal mitochondria of diabetic mice: Possible protection by superoxide dismutase. Investig. Ophthalmol. Vis. Sci..

[113] Madsen-Bouterse S.A., Zhong Q., Mohammad G., Ho Y.S., Kowluru Y.S. (2010). Oxidative damage of mitochondrial DNA in diabetes and its protection by manganese superoxide dismutase. Free Radic. Res..

[114] Kadłubowska J., Malaguarnera L., Wąż P., Zorena K. (2016). Neurodegeneration and Neuroinflammation in Diabetic Retinopathy: Potential Approaches to Delay Neuronal Loss. Curr. Neuropharmacol..

[115] Kowluru R.A., Kanwar M. (2007). Effects of curcumin on retinal oxidative stress and inflammation in diabetes. Nutr. Metabol..

[116] Shi X., Liao S., Mi H., Guo C., Qi D., Li F., Zhang C., Yang Z. (2012). Hesperidin prevents retinal and plasma abnormalities in streptozotocin-induced diabetic rats. Molecules.

[117] Bucolo C., Leggio G.M., Drago F., Salomone S. (2012). Eriodictyol prevents early retinal and plasma abnormalities in streptozotocin-induced diabetic rats. Biochem. Pharmacol..

[118] Chen Y., Sun X.B., Lu H.E., Wang F., Fan X.H. (2017). Effect of luteoin in delaying cataract in STZ-induced diabetic rats. Arch. Pharm. Res..

[119] Zorena K., Kula M., Malinowska E., Raczyńska R., Myśliwiec M., Raczyńska K. (2013). Threshold serum concentrations of tumour necrosis factor alpha (TNFα) as a potential marker of the presence of microangiopathy in children and adolescents with type 1 diabetes mellitus (T1DM). Hum. Immunol..

[120] Bengmark S. (2007). Advanced glycation and lipoxidation end products—Amplifiers of inflammation: The role of food. JPEN J. Parenter. Enter. Nutr..

[121] Vistoli G., De M.D., Cipak A., Zarkovic N., Carini M., Aldini G. (2013). Advanced glycoxidation and lipoxidation end products (AGEs and ALEs): An overview of their mechanisms of formation. Free Radic. Res..

[122] Bahadoran Z., Mirmiran P., Azizi F. (2013). Dietary polyphenols as potential nutraceuticals in management of diabetes: A review. J. Diabetes Metab. Disord..

[123] Da Silva S., Costa J., Pintado M., Ferreira D., Sarmento B. (2010). Antioxidants in the prevention and treatment of diabetic retinopathy—A review. J. Diabetes Metab..

[124] Ito T., Schaffer S.W., Azuma J. (2012). The potential usefulness of taurine on diabetes mellitus and its complications. Amino Acids.

[125] Obrosova I.G., Minchenko A.G., Marinescu V., Fathallah L., Kennedy A., Stockert C.M., Frank R.N., Stevens M.J. (2001). Antioxidants attenuate early up regulation of retinal vascular endothelial growth factor in streptozotocin-diabetic rats. Diabetologia.

[126] Zhang K., Ferreyra H.A., Grob S., Bedell M., Zhang J.J., Ryan S.J. (2013). Diabetic retinopathy: Genetics and etiologic mechanisms. Retina.

[127] Shen J., Bi Y.L., Das U.N. (2014). Potential role of polyunsaturated fatty acids in diabetic retinopathy. Arch. Med. Sci..

[128] Majumdar S., Srirangam R. (2010). Potential of the bioflavonoids in the prevention/treatment of ocular disorders. J. Pharm. Pharmacol..

[129] (2019). World Health Organization Obesity and Overweight: World Health Organization 2018.

[130] Treede R.D., Rief W., Barke A., Aziz Q., Bennett M.I., Benoliel R., Cohen M., Evers S., Finnerup N.B., First M.B. (2019). Chronic pain as a symptom or a disease: The IASP Classification of Chronic Pain for the International Classification of Diseases (ICD-11). Pain.

[131] Bryl A., Mrugacz M., Falkowski M., Zorena K. (2022). The Effect of Hyperlipidemia on the Course of Diabetic Retinopathy-Literature Review. J. Clin. Med..

[132] Tang N., Ma J., Tao R., Chen Z., Yang Y., He Q., Lv Y., Lan Z., Zhou J. (2022). The Effects of the Interaction between BMI and Dyslipidemia on Hypertension in Adults. Sci. Rep..

[133] Lopes de Faria J.B., Silva K.C., Lopes de Faria J.M. (2011). The contribution of hypertension to diabetic nephropathy and retinopathy: The role of inflammation and oxidative stress. Hypertens. Res..

[134] Shrestha A., Suwal R., Adhikari S., Shrestha N., Shrestha B., Khatri B. (2023). Diabetic Retinopathy among Patients with Prediabetes Attending the Outpatient Department of Ophthalmology in a Tertiary Eye Care Centre: A Descriptive Cross-sectional Study. JNMA J. Nepal Med. Assoc..

[135] Kaštelan S., Gverović-Antunica A., Pelčić G., Gotovac M., Marković I., Kasun B. (2018). Refractive Changes Associated with Diabetes Mellitus. Semin. Ophthalmol..

[136] Kostev K., Rathmann W. (2013). Diabetic retinopathy at diagnosis of type 2 diabetes in the UK: A database analysis. Diabetologia.

[137] Kaštelan S., Tomić M., Gverović Antunica A., Ljubić S., Salopek Rabatić J., Karabatić M. (2013). Body mass index: A risk factor for retinopathy in type 2 diabetic patients. Mediat. Inflamm..

[138] Kaštelan S., Salopek Rabatić J., Tomić M., Gverović Antunica A., Ljubić S., Kaštelan H., Novak B., Orešković D. (2014). Body mass index and retinopathy in type 1 diabetic patients. Int. J. Endocrinol..

[139] Price S.A., Gorelik A., Fourlanos S., Colman P.G., Wentworth J.M. (2014). Obesity is associated with retinopathy and macrovascular disease in type 1 diabetes. Obes. Res. Clin. Pract..

[140] Grauslund J., Green A., Sjolie A.K. (2009). Prevalence and 25 year incidence of proliferative retinopathy among Danish type 1 diabetic patients. Diabetologia.

[141] Forga L., Goñi M.J., Ibáñez B., Cambra K., García-Mouriz M., Iriarte A. (2016). Influence of age at diagnosis and time-dependent risk factors on the development of diabetic retinopathy in patients with Type 1 diabetes. J. Diabetes Res..

[142] Henricsson M., Nyström L., Blohme G., Ostman J., Kullberg C., Svensson M., Schölin A., Arnqvist H.J., Björk E., Bolinder J. (2003). The incidence of retinopathy 10 years after diagnosis in young adult people with diabetes: Results from the nationwide population-based Diabetes Incidence Study in Sweden (DISS). Diabetes Care.

[143] Man R.E.K., Sabanayagam C., Chiang P.P.C., Li L.J., Noonan J.E., Wang J.J., Wong T.Y., Cheung G.C.M., Tan G.S.W., Lamoureux E.L. (2016). Differential association of generalized and abdominal obesity with cukrzycotic retinopathy in Asian patients with type 2 diabetes. JAMA Ophthalmol..

[144] Fu S., Zhang L., Xu J., Liu X., Zhu X. (2023). Association between abdominal obesity and diabetic retinopathy in patients with diabetes mellitus: A systematic review and meta-analysis. PLoS ONE.

[145] Uckay G., Ozata M., Bayraktar Z., Erten V., Bingol N., Ozdemir I.C. (2000). Is leptin associated with diabetic retinopathy?. Diabetes Care.

[146] Considine R.V., Sinh M.K., Heiman M.L., Kriauciunas A., Stephens T.W., Nyce M.R., Ohannesian J.P., Marco C.C., McKee L.J., Bauer T.L. (1995). Serum immunore active—Leptin concentrations in normal weight and obese humans. N. Engl. J. Med..

[147] Yang H.S., Choi Y.J., Han H.Y., Kim H.S., Park S.H., Lee K.S., Lim S.H., Heo D.J., Choi S. (2021). Serum and aqueous humor adiponectin levels correlate with diabetic retinopathy development and progression. PLoS ONE.

[148] Elhayany A., Lustman A., Abel R., Attal-Singer J., Vinker S. (2010). A low carbohydrate Mediterranean diet improves cardiovascular risk factors and diabetes control among overweight patients with type 2 diabetes mellitus: A 1-year prospective randomized intervention study. Diabetes Obes. Metab..

[149] Mancini J.G., Filion K.B., Atallah R., Eisenberg M.J. (2016). Systematic review of the Mediterranean diet for long-term weight loss. Am. J. Med..

[150] Sandouk Z., Lansang M.C. (2017). Diabetes with obesity—Is there an ideal diet?. Cleve Clin. J. Med..

[151] Esposito K., Maiorino M.I., Ciotola M., Di Palo C., Scognamiglio P., Gicchino M., Petrizzo M., Saccomanno F., Beneduce F., Ceriello A. (2009). Effects of a Mediterra-nean-style diet on the need for antihyperglycemic drug therapy in patients with newly diagnosed type 2 diabetes: A randomized trial. Ann. Intern. Med..

[152] Estruch R., Martínez-González M.A., Corella D., Salas-Salvadó J., Ruiz-Gutiérrez V., Covas M.I., Fiol M., Gómez-Gracia E., López-Sabater M.C., Vinyoles E. (2006). PREDIMED Study Investigators. Effects of a Mediterranean-style diet on cardiovascular risk factors: A randomized trial. Ann. Intern. Med..

[153] Ferro-Luzzi A., James W.P., Kafatos A. (2002). The high-fat Greek diet: A recipe for all?. Eur. J. Clin. Nutr..

[154] Romaguera D., Norat T., Vergnaud A.C., Mouw T., May A.M., Agudo A., Buckland G., Slimani N., Rinaldi S., Couto E. (2010). Mediterranean dietary patterns and prospective weight change in the participants of the EPIC-PANACEA project. Am. J. Clin. Nutr..

[155] Steffen L.M., Van Horn L., Daviglus M.L., Zhou X., Reis J.P., Loria C.M., Jacobs D.R., Duffey K.J. (2014). A modified Mediterranean diet score is associated with a lower risk of incident metabolic syndrome over 25 years among young adults: The CARDIA (Coronary Artery Risk Development in Young Adults) study. Br. J. Nutr..

